# Alpha-Synuclein-Specific Naturally Occurring Antibodies Inhibit Aggregation In Vitro and In Vivo

**DOI:** 10.3390/biom12030469

**Published:** 2022-03-18

**Authors:** Anne K. Braczynski, Marc Sevenich, Ian Gering, Tatsiana Kupreichyk, Emil D. Agerschou, Yannick Kronimus, Pardes Habib, Matthias Stoldt, Dieter Willbold, Jörg B. Schulz, Jan-Philipp Bach, Björn H. Falkenburger, Wolfgang Hoyer

**Affiliations:** 1 Department of Neurology, RWTH University Hospital, 52074 Aachen, Germany; abraczynski@ukaachen.de (A.K.B.); phabib@ukaachen.de (P.H.); jschulz@ukaachen.de (J.B.S.); j_pbach@yahoo.de (J.-P.B.); 2 Institut für Physikalische Biologie, Heinrich-Heine University Düsseldorf, 40225 Düsseldorf, Germany; tatsiana.kupreichyk@hhu.de (T.K.); emil.agerschou@hhu.de (E.D.A.); m.stoldt@fz-juelich.de (M.S.); d.willbold@fz-juelich.de (D.W.); 3 Institute of Biological Information Processing (IBI-7: Structural Biochemistry), Forschungszentrum Jülich, 52428 Jülich, Germany; m.sevenich@fz-juelich.de (M.S.); i.gering@fz-juelich.de (I.G.); 4 Priavoid GmbH, 40225 Düsseldorf, Germany; 5 Department of Geriatric Medicine, University Hospital Essen, University Duisburg-Essen, 47057 Duisburg, Germany; yannickk89@web.de; 6 Institute of Biochemistry and Molecular Immunology, Medical Faculty, RWTH Aachen University, 52074 Aachen, Germany; 7 JARA-Institute Molecular Neuroscience and Neuroimaging, Jülich Aachen Research Alliance, FZ Jülich and RWTH University, 52428 Jülich, Germany; 8 Department of Neurology, University Hospital Carl Gustav Carus, 01307 Dresden, Germany

**Keywords:** aggregation, Parkinson’s disease, intravenous immunoglobulins (IVIG), naturally occurring antibodies, alpha-synuclein

## Abstract

Parkinson’s disease (PD) is associated with motor and non-motor symptoms and characterized by aggregates of alpha-synuclein (αSyn). Naturally occurring antibodies (nAbs) are part of the innate immune system, produced without prior contact to their specific antigen, and polyreactive. The abundance of nAbs against αSyn is altered in patients with PD. In this work, we biophysically characterized nAbs against αSyn (nAbs-αSyn) and determined their biological effects. nAbs-αSyn were isolated from commercial intravenous immunoglobulins using column affinity purification. Biophysical properties were characterized using a battery of established in vitro assays. Biological effects were characterized in HEK293T cells transiently transfected with fluorescently tagged αSyn. Specific binding of nAbs-αSyn to monomeric αSyn was demonstrated by Dot blot, ELISA, and Surface Plasmon Resonance. nAbs-αSyn did not affect viability of HEK293T cells as reported by Cell Titer Blue and LDH Assays. nAbs-αSyn inhibited fibrillation of αSyn reported by the Thioflavin T aggregation assay. Altered fibril formation was confirmed with atomic force microscopy. In cells transfected with EGFP-tagged αSyn we observed reduced formation of aggresomes, perinuclear accumulations of αSyn aggregates. The results demonstrate that serum of healthy individuals contains nAbs that specifically bind αSyn and inhibit aggregation of αSyn in vitro. The addition of nAbs-αSyn to cultured cells affects intracellular αSyn aggregates. These findings help understanding the role of the innate immune systems for the pathogenesis of PD and suggest that systemic αSyn binding agents could potentially affect neuronal αSyn pathology.

## 1. Introduction

Parkinson’s disease (PD) is the second-most common neurodegenerative disorder. The characteristic pathological changes consist of dopaminergic neuronal loss in the substantia nigra, gliosis, and intraneuronal alpha-synuclein (αSyn) pathology, comprising Lewy bodies and Lewy neurites [1]. αSyn is the predominant protein in Lewy body inclusions [2]. αSyn pathology spreads along anatomical connections with gut and olfactory bulb constituting putative entry points into the nervous system [3,4]. The theory of multiorgan spreading of αSyn along the “gut–brain-axis” is supported by a large body of evidence from patient cohorts and animal experiments [5,6,7,8,9,10]. Consistent with the origin of αSyn pathology in the periphery, αSyn deposits were also found in glands [11], skin [12,13], and gastrointestinal tissue [14,15].

αSyn is a natively unfolded protein of 140 amino acids encoded by the gene *SNCA* and localized in presynaptic terminals [16]. αSyn consists of three domains: The N-terminus (aa 1–60) contains the positions of the six mutations that cause hereditary PD and sites for post-translational modifications [17,18]. The hydrophobic non-amyloid component (NAC) region (aa 61–95) is important for aggregation, oligomerization, and for building β–sheet containing structures including fibrils [19]. The C-terminal domain (aa 96–140) is proline- and aspartate/glutamate-rich, contains phosphorylation sites [20,21], and is the epitope to most antibodies tested as therapeutic strategies against PD [22]. 

The discovery of αSyn pathology in PD led to a plethora of immunization studies [22,23]. Different animal models have consistently illustrated the potential of passive [24,25,26] and active [27,28] immunization to reduce brain αSyn pathology and improve motor outcome. Clinical studies are on their way [22,29]. 

Naturally occurring antibodies (nAbs) are part of the innate immune system, produced without contact to the specific antigen they recognize and polyreactive [30]. The majority of reported nAbs are IgM and IgG [31] and seem to play a role in neurodegenerative disorders [32,33], but also in other diseases, such as chronic inflammatory diseases or atherosclerosis [34]. Although their mechanism of action is fairly unclear, nAbs have been shown to identify apoptotic debris and initiate its phagocytic removal [34,35]. There are also nAbs against αSyn (nAbs-αSyn) [36]. Their abundance is altered in PD patients [37,38,39]. The field is, however, still uncertain about the direction of change of nAbs-αSyn in PD. Several groups described decreased nAbs-αSyn in the serum of patients with PD as compared to healthy controls [36,37,40]. Yet, other groups described no changes [38], or even increased levels [39,41,42,43,44]. Interestingly, nAbs-αSyn are already present in early childhood with levels comparable to healthy adult controls [45]. The binding of nAbs to αSyn monomers, oligomers, and fibrils could be confirmed using dot blot, surface plasmon resonance (SPR) and enzyme-linked immunosorbent assay (ELISA) [26,46]. nAbs-αSyn purified from commercial intravenous immunoglobulins (IVIG) using αSyn oligomers inhibit aggregation of αSyn as reported by thioflavin T (ThT) fluorescence [26].

Intracellular αSyn aggregates are transported towards the perinuclear aggresome and degraded by autophagy [47,48]. In this process, the aggresome is a steady-state structure that grows whenever generation and transport of aggregates exceeds the capacity of autophagic clearance [49]. αSyn affects cellular homeostasis by different mechanism and nAbs-αSyn attenuated toxicity resulting from αSyn oligomers in N2a cells [26].

Given the importance of better understanding PD pathogenesis and providing effective therapies for patients, reproducing findings is of particular importance in Translational Neurosciences [50]. Basic research lately could not translate into the development of therapeutics to cure diseases despite promising findings; the reasons for this reproducibility crisis are numerous and reviewed in [50]. We, therefore, characterized in this study nAbs against αSyn derived from IVIG preparations that are commercially available in Germany, using a standard battery of Dot blot, ELISA, SPR, and biophysical methods including Thioflavin T (ThT) aggregation assay and Atomic Force Microscopy (AFM). Furthermore, we determined their effects in a cellular model of αSyn pathology [51].

## 2. Materials and Methods

### 2.1. Gravity Flow Affinity Purification of nAbs

nAbs-αSyn were isolated from IVIG as previously described [46]. Briefly, we used purified intravenous immunoglobulin G (IgG) (Gamunex 10%, 100 mg/mL, Grifols, Barclona, Spain). Then, 96% of protein represents normal human IgG (IgA < 0.2 mg (0.02%); IgM < 0.1 mg (0.01%)). IgG subclasses are fully represented (IgG 1, 65%; IgG 2, 30%; IgG 3, 3%; IgG 4, 2%). Recombinant αSyn (rPeptides, Bogart, GA, USA) was coupled to aldehyde-activated agarose support (Aminolink Plus Coupling Resin; Thermo Scientific, Waltham, MA, USA). A total of 20 mL of 2.5% IVIG was added to the column in fractions of 3.5 mL, followed by incubation at room temperature for 60 min per fraction. After washing with PBS, the bound antibodies were eluted from the column with 10 × 0.5 mL 0.1 M glycine buffer, and pH 2.8. Each fraction was collected in a microreaction tube containing 35 μL 1 M Tris–HCl, pH 9. IVIg depleted of nAbs-αSyn, termed flowthrough (FT), was also collected and used as a negative control in experimental settings. To maintain the integrity of the antibodies, a neutral pH was adjusted immediately after elution by adding the appropriate amount of Tris-HCI or glycine buffer. nAbs-αSyn of several purification rounds were concentrated with Vivaspin concentration device (Vivaspin 20, 5 k, Sartorius, Goettingen, Germany) according the manufacturer´s instructions. A total of 25 purification batches resulted in ca. 250 µL of nAbs-aSyn at a concentration of ca. 180 µM nAbs-αSyn. Protein concentration was measured with the DC Protein Assay (Bio-Rad, Dreieich, Germany) in a 96-well-plate (Falcon, Corning, Glendale, AZ, USA) against a standard calibration curve. Absorbance at 756 nM was measured after 15 min incubation on a microplate reader (Infinite 200 PRO Microplate Reader, Tecan, Crailsheim, Germany).

### 2.2. Dot Blot

**Figure 1:**
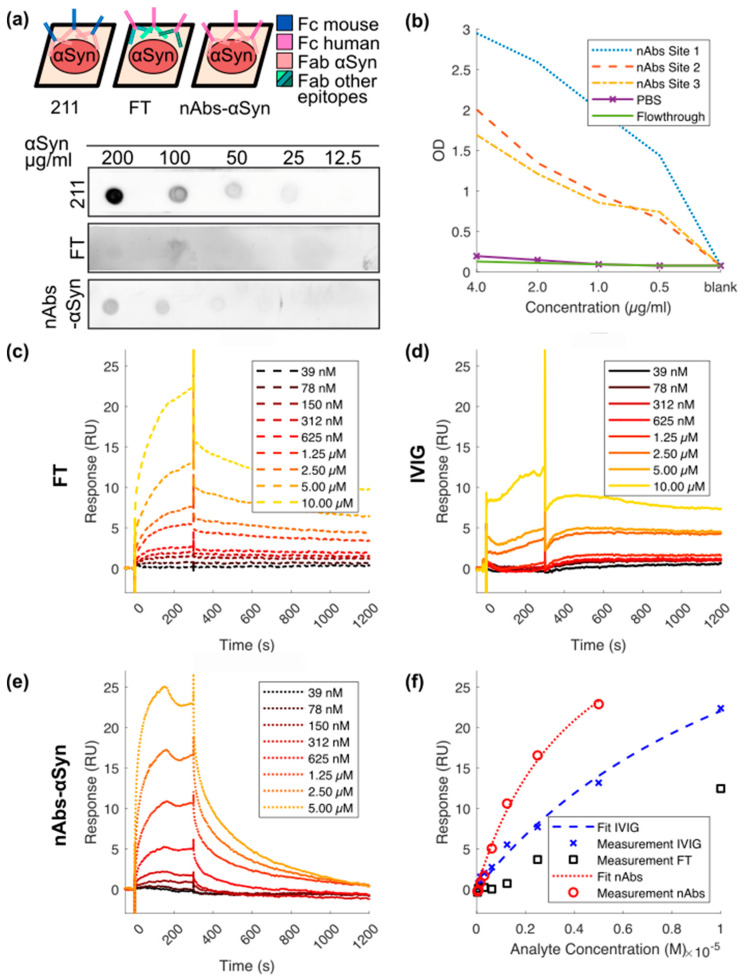
Binding characteristics of nAbs-αSyn in dot blot, ELISA, and SPR. (**a**) nAbs-αSyn recognize αSyn on a dot blot: αSyn at different concentrations were spotted on a nitrocellulose membrane and then probed with flow through and nAbs-αSyn. Anti-αSyn antibody was used as a control. Although the control antibody (211) is a mouse antibody, FT and nAbs-αSyn are of human origin (see sheme). Each dot blot requires a different secondary antibody (either anti-mouse or anti-human) (**b**) nAbs-αSyn recognize αSyn in an ELISA: nAbs-αSyn from three different project sites were tested in an ELISA, to determine their capacity to bind to αSyn in vitro. (**c**–**f**) nAbs-αSyn recognize αSyn in SPR: SPR curves of nAbs-αSyn (**e**) binding to recombinant αSyn. IVIG (**c**) and FT (**d**) were used as controls. A concentration range of 39 nM–5 µM of nAbs-αSyn was applied in SPR. The binding affinity was determined from three replicates.

The indicated amounts of recombinant αSyn were spotted onto nitrocellulose membranes (Amersham Protan 0.2 µM, Chalfont St. Giles, Great Britain), dried for 15 min and blocked for 1 hour at 20 °C once in RotiBlock (Carl Roth, Karlsruhe, Germany). Each dot blot was incubated with either 0.2 µg/mL nAbs-αSyn, FT or anti-αSyn antibody (1:2000, Syn 211, Thermo Scientific, Rockford, USA) over night at 4 °C and washed 3 times with Tris buffered saline with 0.05% Tween-20 (TBS-T) for 30 min. As secondary antibody, horse reddish peroxidase (HRP) coupled to anti-human IgG antibodies (Peroxidase conjugated Affinity Pure Anti-Human IgG, Jackson Immunoresearch, Ely, United Kingdom) was used to detect the antibodies of human source (nAbs-αSyn and FT). Anti-mouse antibodies (Peroxidase conjugated Affinity Pure Goat anti-mouse IgG H + L, Jackson Immunoresearch, Ely, United Kingdom) were used to detect the anti-αSyn antibody. Blots were developed with Super Signal West Femto Max Sensitivity Substrate (Thermo Scientific, Waltham, MA, USA) on Bio-Rad ChemiDoc. (Bio-Rad, Munich, Germany). The experimental setup is schematically depicted in Figure 1a.

### 2.3. Enzyme-Linked Immunosorbent Assay (ELISA)

Wells of high-binding 96-well ELISA plates (Immulon Microtiter Plates 2HB “U” Bottom, Thermo Scientific, Waltham, MA, USA ) were coated with 50 µL of 3 µg/mL recombinant αSyn (Analytic Jena, Jena, Germany) in phosphate-buffered saline (PBS). After 24 h at 4 °C, wells were blocked with 180 µL blocking buffer (1× Rotiblock + 0.1% Tween-20, Carl Roth, Karlsruhe, Germany) for additional 24 h at 4 °C. Antibodies were added to the wells in a series of decreasing concentrations starting from 4 µg/ml. Plates were incubated with samples for 1 h at 20 °C and shaken on an orbital platform shaker (Unimax 1010, Heidolph, Schwabach, Germany) at 100 rpm. Afterwards, 50 µL/well biotinylated goat anti-human detection antibody was added to the wells (Dianova GmbH, Hamburg, Germany, 1:20,000 in blocking buffer). After 1 h at 20 °C and 100 rpm, the samples were incubated with 50 µL/well of streptavidin-peroxidase (Streptavidin-HRP, R&D Systems, Minneapolis, MN, USA, 1:200 in blocking buffer) and shaken for 20 min at 20 °C and 100 rpm. The assay was developed using 50 µL/well Tetramethylbenzidine (TMB) (Merck, Darmstad, Germany). After 20 min, the reaction was stopped by adding 25 µL/well of 5% sulfuric acid (Carl Roth, Karlsruhe, Germany). Absorbance at 450 nM was measured using a microplate reader (Infinite 200 PRO Microplate Reader, Tecan, Crailsheim, Germany). All incubation steps were performed in the dark. After each step up to and including TMB addition, the wells were washed three times with 300 µL 1× PBS + 0.05% Tween-20 using an Amersham Biotrak II Plate Washer (GE Healthcare, Chicago, IL, USA).

### 2.4. Surface Plasmon Resonance (SPR)

SPR measurements were performed using a Biacore T200 instrument (GE Healthcare, GE Healthcare, Chicago, IL, USA) at 25 °C with PBS/0.05% Tween-20, pH 7.4 as running buffer. For preparation of the flow cells, a CM5 sensor chip (GE Healthcare, Chicago, IL, USA) was activated with 1-ethyl-3-(3-dimethylaminopropyl) carbodiimide (EDC)/N-hydroxysuccinimide (NHS) (0.2 M/0.05 M). Recombinant full-length αSyn (200 ng/mL) was diluted in 10 mM sodium acetate, pH 4.0. It was immobilized to a final level of 125 reactive units (RU), and the flow cell was deactivated with 1 M ethanolamine-HCl, pH 8.5. A reference flow cell was activated and deactivated only. Afterwards, IVIG, FT and nAbs-αSyn at concentrations ranging from 39 nM to min. 5 μM were injected once followed by 5 cycles of regeneration with 5 M urea. All samples were injected over the flow cells for 300 s, followed by a dissociation step of 900 s. The sensorgrams were double referenced. Evaluation was performed by plotting the respective response levels against the applied nAbs-αSyn concentrations in a steady-state fit. For data evaluation, the sensorgrams were calculated in the Biacore T200 Evaluation Software 3.1 (version 3.1, proprietary software of GE Healthcare).

### 2.5. Thioflavin T Aggregation Assay

ThT aggregation assays were conducted as described before [52] in Corning half area 96-well plates with non-binding surface (Corning No. 3881, Glendale, AZ, USA). For the assays starting from monomeric αSyn, 25 μM of wild type (WT) αSyn was used. Aggregation assays were run for 60 h with measurement of ThT fluorescence every 20 min (λEx = 450 nm, bandwidth 5 nM; λEm = 482 nM, bandwidth 10 nM) with 15 s of orbital shaking before the measurement in a plate reader (BMG Clariostar Plus, Ortenberg, Germany). The assays were conducted at 37 °C in 25 mM K-phosphate buffer at pH 7.3, 100 mM KCl, 1 mM MgCl_2_, 10 μM ThT, and 0.05% NaN_3_, reflecting intracellular potassium and magnesium concentration, as well as intracellular pH and ionic strength. A glass ball of 2.85–3.45 mm size was added per well to improve mixing. Per well, a sample volume of 125 μL sample was used. For evaluation, the mean of triplicate measurements was referenced to the highest fluorescence of 25 μM WT αSyn.

### 2.6. Atomic Force Microscopy

AFM images were taken with a Nanowizard 3 atomic force microscope (JPK, Berlin, Germany) in intermittent contact mode (AC mode) in air, using silicon cantilever and tip (OMCL-AC160TS-R3, Olympus, Hamburg, Germany) with a typical tip radius of 9 ± 2 nM, a force constant of 26 N/m and a resonance frequency of approximately 300 kHz. The images derive from a manual observer-blind estimation, and provide qualitative characteristics. The image processing was performed using JPK data processing software (version spm-5.0.84): for each of the presented height profiles, a polynomial fit was subtracted from each scan line first independently and then using limited data range. For the sample preparation, solutions containing fibrils were diluted to a concentration of 1 µM (in monomer equivalents) in water and 5 μL samples of the diluted solution were deposited on freshly cleaved muscovite mica and left to dry for at least 30 min. The samples were carefully washed with 50 µL of double distilled H_2_O and then dried again with a stream of N_2_ gas before imaging.

### 2.7. Cell Culture

HEK293T (RRID: CVCL_0063) cells were cultured in Dulbecco’s modified Eagle’s medium supplemented with 10% fetal calf serum and 1% penicillin-streptomycin. ‘HEK’ cells are listed in version 8.0 of the Database of Cross-Contaminated or Misidentified Cell Lines. Our HEK293T cells were validated in November 2017 by analysis of 21 genetic loci (Promega, Madison, WI, USA, PowerPlex 21 PCR Kit carried out by Eurofins Medigenomix Forensik, Ebersberg, Germany). Cells were plated on poly-L-lysine-coated glass cover slips or on plastic plates. Transfection was performed 18–20 h after plating using Metafectene (Biontex Laboratories, Munich, Germany). Unless noted otherwise, subsequent analysis was completed 24 h after transfection.

### 2.8. LDH Release

CytoTox 96 Non-Radioactive Cytotoxicity Assay (Promega, Madison, WI, USA) was performed according to the manufacturer’s protocol to measure release of lactate dehydrogenase (LDH) as a marker of cellular viability, as described before [53]. HEK293T were seeded on a 96-well plate 48 h prior to treatment with nAbs-αSyn. After 1 h/3 h/6 h/24 h incubation time, 50 μL of each well was transferred to a fresh 96-well plate. In addition to a no-treatment-cell control, a no-cell control, and a control containing HEK293T lysed with Triton X-100 were used. CytoTox 96 Reagent (Promega, Madison, WI, USA) was added to each well, and the absorbance was recorded at 490 nM by Infinite M200 (Tecan, Männedorf, Switzerland). Data are presented as a percentage of maximum LDH release (100%), which was determined by HEK293T lysed with 1% Triton X-100.

### 2.9. Metabolic Activity Assay

To assess metabolic activity a CellTiter-Blue (CTB) Cell Viability Assay was performed according to manufacturer’s protocol as described before [53]. HEK293T were seeded on a 96-well plate 48 h prior to treatment with nAbs-αSyn. After 1 h/3 h/6 h/24 h incubation time, CellTiter-Blue Reagent (Promega, Madison, WI, USA) was added to each well. After 2.5–3 h, a color switch (reduction in resazurin) was observed and fluorescence was recorded at 560Ex/590Em by Infinite M200 (Tecan, Männedorf, Switzerland).

### 2.10. Plasmids

A53T-αSyn was flexibly tagged with EGFP by using the interaction of a PDZ domain (post synaptic density protein (PSD95), Drosophila disc large tumor suppressor (Dlg1), and zonula occludens-1 protein (zo-1)) with its six amino acid-binding motifs. PDZ-EGFP was coexpressed with αSyn to which a six amino acid PDZ binding domain was added at the C-terminus [51].

### 2.11. Microscopy of Fixed Cells

Analysis of cells expressing EGFP-tagged αSyn was carried out as previously described [51,54]. For the classification of EGFP distribution patterns, cells were grown and transfected on coverslips. Then, 24 hours after transfection, cells were washed three times in cold PBS and fixed with paraformaldehyde solution (PFA, 4% paraformaldehyde, 5% sucrose in PBS) for 10 min. Coverslips were mounted with Fluoromount g (Cat# 0100-01 Southern Biotech, Birmingham, AL, USA). Using standard fluorescence microscopy (BX51 microscope, 60× oil immersion objective, NA 1.35, Olympus, Hamburg, Germany) focal depth was sufficient to see the entire cell in focus. The EGFP distribution pattern was classified manually into the following categories: ‘homogenous’, ‘one aggresome’ (large, round, perinuclear aggregate), ‘aggresome and aggregates’ and ‘unhealthy’ (round, condensed cells). Z-Stacks were not aquired. A blinded investigator classified at least 100 cells per coverslip. In each experiment, three coverslips were evaluated per experimental group and the results averaged by calculating mean. Depicted graphs summarize data (mean standard error of the mean (SEM)) from three independent experiments with ‘n’ equal to the number of independent experiments summarized.

### 2.12. Statistics

The study was not pre-registered. For assignment of experimental groups, no special randomization methods were employed. Sample sizes were determined by past experience and not by a statistical sample size calculation (Figure 3: at least 100 cells were analyzed). For statistical analysis and data presentation, we used GraphPad Prism 5.0 and 6.0 (RRID: SCR_002798, GraphPad Software, LaJolla, CA, USA). Graphs represent mean SEM. The tests used for comparison in each graph are noted in the figure legend (Figure 3: one-way ANOVA). The experiments were replicated independently at least three times with three replicates, representative graphs are shown. *P* < 0.05 was considered significant. In graphs, p-values are depicted as *p < 0.05, **p < 0.01, ***p < 0.001, ****p < 0.0001.

## 3. Results

### 3.1. Experimental Work

#### 3.1.1. Binding Characteristics of nAbs-αSyn

nAbs-αSyn were isolated from commercial human IVIG preparations using gravity flow affinity purification as described previously [46]. nAbs-αSyn are not very abundant, so several purification batches were pooled to obtain a sufficient amount of nAbs-αSyn protein. The flow through (FT) of the affinity purification was used as negative control.

First, we analyzed binding of nAbs-αSyn to monomeric αSyn protein by dot blot. In a dot blot, the bait protein is immobilized to a solid surface (nitrocellulose) and incubated with the prey protein (the indicated probes containing antibodies) in the fluid phase (as depicted in the sheme, Figure 1a). Dependent on the species of the prey protein, the detection system varies. We observed concentration-dependent immunoreactivity against Fc termini using either a commercial αSyn antibody (Figure 1a, top) or nAbs-αSyn (Figure 1a, bottom), but not using FT (Figure 1a, middle). Specific binding of nAbs-αSyn to monomeric αSyn protein was confirmed in ELISA measurements (Figure 1b). αSyn was detected by all nAbs-αSyn with different avidities even at low concentrations of nAbs-αSyn (0.5 μg/mL). Furthermore, nAbs were significantly higher in their avidity in comparison to the reference control FT or PBS, resulting in a higher binding signal. In SPR measurement nAbs-αSyn interacted strongly with αSyn. Due to the polyreactive nature of nAbs-αSyn, the dissociation constant (*K*_D_) was not determined from the binding kinetics but from a steady state fit, yielding a value of *K*_D_ = (4.22 ± 0.95) × 10^−6^ M. nAbs-αSyn showed a higher affinity than IVIG and FT (Figure 1c–f), confirming that binding agents with specificity for αSyn were enriched during nAbs-αSyn preparation. nAbs-αSyn binding to αSyn could be confirmed with all three methods.

#### 3.1.2. nAbs-αSyn Interfere with αSyn Amyloid Formation and Alter αSyn Fibril Morphology as Visualized with Atomic Force Microscopy

**Figure 2:**
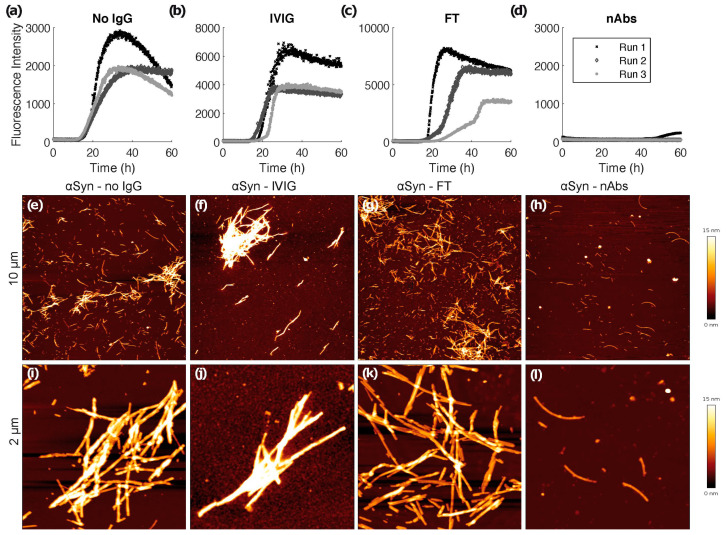
ThT fibrillation assay and atomic force microscopy. De novo fibrillation of 25 µM αSyn in the absence (**a**) and presence of 0.1 µM IVIG (**b**), FT (**c**) and nAbs-αSyn (**d**) followed by ThT fluorescence. Three replicates are shown per condition. At the end of the 60 h time course, samples were imaged via AFM (**e**–**l**). Representative overview images of the samples showing 10 × 10 µm areas (**e**–**h**) and 2 × 22 µM close-ups (**i**–**l**).

We performed a ThT fibrillation assay to assess de novo αSyn amyloid formation in absence and presence of nAbs-αSyn. In the absence of nAbs-αSyn, the typical sigmoidal kinetics of nucleated polymerization of αSyn monomers into amyloid fibrils was observed (Figure 2a). Although there was some variability between experimental repeats in the shapes of the time traces and in the final ThT fluorescence intensities, the lag-time of aggregation, i.e., the time after which amyloid formation is detectable by ThT fluorescence for the first time, was quite uniform with a value of ~15 h. FT or IVIG at a protein concentration of 0.1 µM were not able to interfere with amyloid formation of 25 µM αSyn as assessed by lag-times and ThT fluorescence intensities (Figure 2b,c). In contrast, in the presence of 0.1 µM nAbs-αSyn there was either no increase in ThT fluorescence during the 60 h experiment, or a weak fluorescence increase after a prolonged lag-time (Figure 2d). We corroborated the effects seen in the fibrillation assay by AFM. Samples from the fibrillation assay were prepared for AFM, and representative images are shown in Figure 2e–l. A substantially lower amount of fibrils was observed in images of nAbs-αSyn-containing samples (Figure 2h,l) compared to samples that were either untreated (Figure 2e,i) or supplemented with IVIG (Figure 2f,j) or FT (Figure 2g,k). Moreover, the αSyn fibrils in the sample treated with nAbs-αSyn appeared to be thinner and exhibited a lower tendency to cluster than those in the other samples (Figure S1).

#### 3.1.3. Treatment with nAbs-αSyn Decreases Occurrence of Aggresomes in Cells

**Figure 3:**
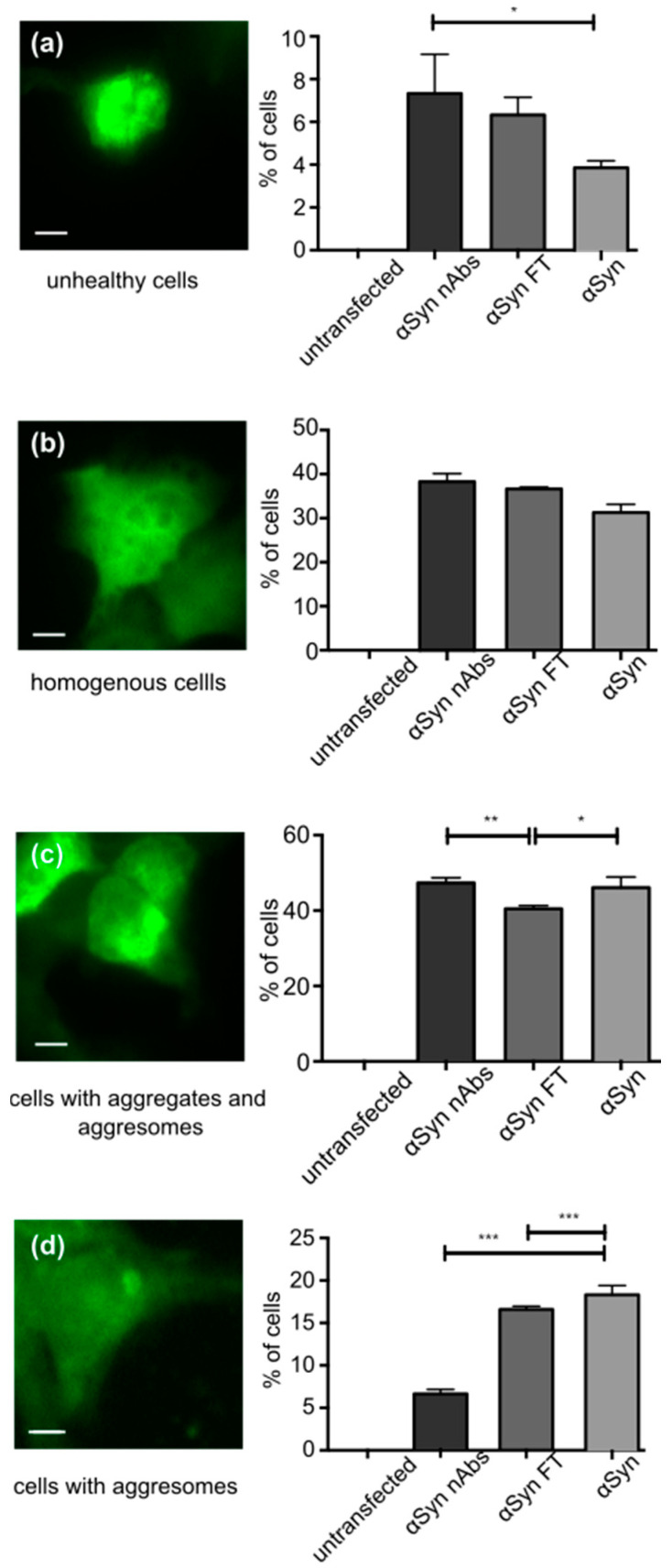
Effect of nAbs-αSyn on GFP-tagged αSyn in HEK293T cells. HEK293T cells were transfected with αSyn flexibily tagged by GFP, grown for 24 h and then treated with nAbs-αSyn for another 24 h. (**a**–**d**) First row, example images of cells in the following categories into which cells were manually classified: Unhealthy cells (**a**), cells with homogenous GFP fluorescence (**b**), cells with aggresomes and aggregates (**c**), cells with aggresomes (**d**). Scale bar represents 5 µm. (**a**–**d**) Second row, quantification of *n* = 3 experiments with at least 100 cells classified for each condition. FT: flow through. Bars represent p values from three replicates as calculated with one-way ANOVA. * *p* < 0.05, ** *p* < 0.01, *** *p* < 0.001.

As a first test, we tested whether nAbs-αSyn affected cell viability in untransfected HEK293T cells. We observed no alteration in cell viability as assessed for LDH-release or metabolic activity (CTB assay) (Figure S2). Next, we tested wether nAbs-αSyn added to the culture medium can affect the behavior of intracellular αSyn. We transfected HEK293T cells with EGFP-tagged αSyn as previously reported [51,54,55].The aSyn construct is modified only by addition of 6 amino acids to the C-terminus of aSyn. This is a small modification given that the C-terminus is relatively flexible. We, therefore, assume that the aggregation properties are not changed much. This aSyn construct is tagged by EGFP by the interaction of this 6 amino acid PDZ binding motif with its PDZ domain, which is coexpressed fused to EGFP. We cannot exclude that even the reversible binding of aSyn to PDZ-EGFP changes the aggregation properties, but to lesser extent than a direct fusion of aSyn-EGFP would alter the aggregation properties. In addition the EGFP-αSyn transfection, cells were treated with nAbs-αSyn or FT for 24 h. Based on the subcellular GFP distribution, we classified cells as unhealthy cells, cells with homogenous GFP fluorescence and cells with one aggresome or aggresome and aggregates. nAbs-αSyn significantly reduced the number of cells with a visible aggresome (Figure 3d, quantified in Figure 3h). At the same time, the number of cells with small aggregates was higher in cells treated with nAbs-αSyn than in cells with FT (Figure 3g). We also observed more unhealthy cells with nAbs-αSyn treatment than without any treatment (Figure 3e), but since there was no significant difference between cells treated with nAbs and FT, we consider this effect nonspecific.

## 4. Discussion

In this work, we characterized nAbs for αSyn that were purified from commercial immunoglobulin preparations using biophysical methods and cultured cell lines. nAbs-αSyn bound αSyn specifically and were able to affect the subcellular distribution of αSyn, suggesting that they can be taken up by cells.

nAbs-αSyn can be isolated from IVIG preparations, as we and others have demonstrated [26,45,46]. IVIG are typically derived from young, healthy plasma donors without any past medical history. Although it cannot be formally excluded that some donors have a neurodegenerative disease or will develop one in the near future, the more likely hypothesis is that this is not the case. The presence of nAbs-αSyn in IVIG preparation, therefore, suggests that nAbs-αSyn are ubiquitously present in healthy individuals. Indeed, nAbs-αSyn were also observed in serum samples of children [45]. nAbs-αSyn, thus, are part of the innate immune system and not an acquired response to the presence of αSyn pathology.

nAbs-αSyn were isolated from IVIG using gravity flow affinity purification, i.e., they are defined by binding recombinant αSyn protein. In order to obtain an estimate of the affinity of nAbs-αSyn for αSyn, we measured the association and dissociation by SPR. Although the Langmuir model used previously to calculate the *K*_D_ [46] is perfectly suitable for monoclonal reactants, we employed a steady state fit to better model the polyreactive nature of nAbs. The obtained apparent *K*_D_ of 4 µM is an order of magnitude higher than the value previously determined from the binding kinetics [46]. It should be noted, however, that the obtained value results from the superposition of binding of different molecular species that might substantially differ in their affinities.

Binding of nAbs-αSyn to αSyn is further confirmed by the fact that they inhibit aggregation of αSyn in vitro (Figure 3). Importantly, this effect is neither observed with the standard IVIG preparation, which contains only small quantities of nAbs-αSyn, nor with the FT. nAbs-αSyn are polyreactive, so they likely bind to different epitopes on the αSyn protein. Our data do not reveal whether all nAbs-αSyn species contribute equally to the inhibition of αSyn aggregation, or whether some species are particularly potent. In this context, it is interesting to note that antibodies and other binding proteins to a variety of αSyn sequence regions, including N-terminus, NAC region, and C-terminus were shown to inhibit aggregation [55,56,57,58,59,60,61,62].

Our study focuses on the behavior of nAbs-αSyn. In addition, more than 50 non-naturally occurring antibodies against αSyn have been established and described, including engineered antibodies and antibody fragments to target αSyn for various purposes in the field of research, diagnostics, therapeutic approaches, and biomarkes [63]. These antibodies recognize epitopes that are as versatile as the protein αSyn itself: They do not only recognize different αSyn sequences (see above), different αSyn conformations (oligomers, fibrils), and, also, different post-translational modifications [63,64,65]. The anti-aggregation effect of many of these antibodies was shown in various studies [23,24,25,26,27,28].

With regard to the potential mechanism of aggregation inhibition, we have shown here that binding of α-synuclein monomers can lead to potent, substoichiometric inhibition of aggregation, in the case when the 1:1 complex of α-synuclein and binding protein acts as an inhibitor of nucleation processes [57]. Moreover, nAbs-αSyn may potently interfere with aggregation also by interacting with higher-order oligomeric or fibrillar species [26]. Interestingly, we observe in AFM that nAbs-αSyn not only reduce the amount of fibrillar aggregates, but also decrease fibril clustering. This might indicate that nAbs-αSyn alter the properties of αSyn fibril surfaces. However, the reduced formation of higher-order aggregate clusters might also simply stem from the lower fibril concentration in presence of nAbs-αSyn.

In order to test whether inhibition of αSyn aggregation observed in vitro translates to an effect in living cells, we used the HEK293T cell line. Adding nAbs-αSyn to these cells did not alter viability (Figure S1). We analyzed the effect of nAbs-αSyn addition to the cell culture medium on the subcellular distribution of EGFP-tagged αSyn (Figure 3). nAbs-αSyn specifically decrease the fraction of cells with a visible aggresome. Aggresomes are formed by retrograde transport of protein aggregates to the microtubule organizing center by dynein motors and cleared by autophagy [47,49]. We cannot know from our findings whether nAbs-αSyn reduce aggresome formation or increase clearance, but nAbs-αSyn are certainly able to affect intracellular processing of αSyn aggregates. These findings should be confirmed in future studies. Additional approaches to study this issue are immunostaining for αSyn [66], pS129 αSyn [64,65], or p62 [67,68]. Analytically, these stainings followed by the puncta per cell quantification together with integrated intensity would nicely complement the EGFP visualization of αSyn and grant clearer insights into its subcellular distribution.

## 5. Conclusions

In summary, we confirmed the presence of nAbs that specifically bind αSyn monomers in IVIG preparations obtained from healthy donors. These nAbs alter αSyn aggregation and fibril conformation in vitro and affect intracellular αSyn processing. Collectively, these findings are consistent with an involvement of the innate immune system in the pathogenesis of PD and with potential therapeutic effects of antibody preparations in patients with PD. Yet, several important questions remain unsolved, such as the effects of nAbs on microglia and astrocytes in the brain, the role of peripheral as opposed to central αSyn deposits for PD pathogenesis, and the species or epitopes of αSyn that are targeted by nAbs.

## Data Availability

Not applicable.

## Supplementary Materials

The following supporting information can be downloaded at: https://www.mdpi.com/article/10.3390/biom12030469/s1, Figure S1: Height distribution of αSyn aggregates formed in presence or absence of nAbs. Figure S2: nAbs-αSyn do not affect viability of untransfected HEK293T cells.
