# Supplementary material for: Alpha-Synuclein-Specific Naturally Occurring Antibodies Inhibit Aggregation In Vitro and In Vivo
Source: Biomolecules. 2022 Mar 18;12(3):469. doi: 10.3390/biom12030469 (PMC8946620; doi:10.3390/biom12030469)
Supplement: Supplementary file 1 [file biomolecules-12-00469-s001.zip › biomolecules-1617665-supplementary.pdf]

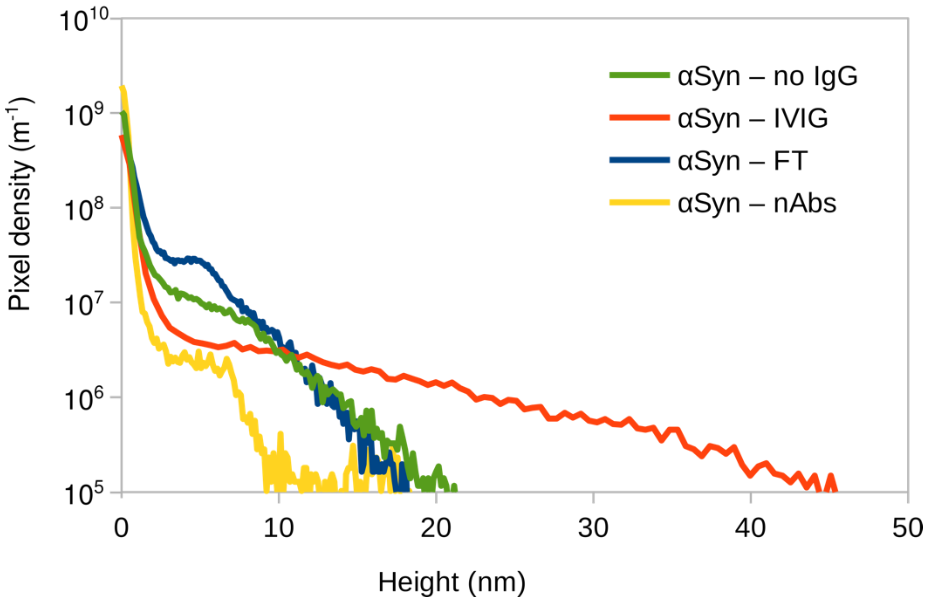

Supplementary Figure S1: Height distribution of αSyn aggregates formed in presence or absence of nAbs

Height distribution histograms extracted from the AFM images taken after the ThT-fluorescence assay (Figure 2 e-h). The highest number of counts correspond to the plane background surface around 0 nm, further distinct shoulder or peak represent the aggregate heights. Extended shoulder of in αSyn-IVIG sample can be attributed to the clustering and according overlay of the fibrils.

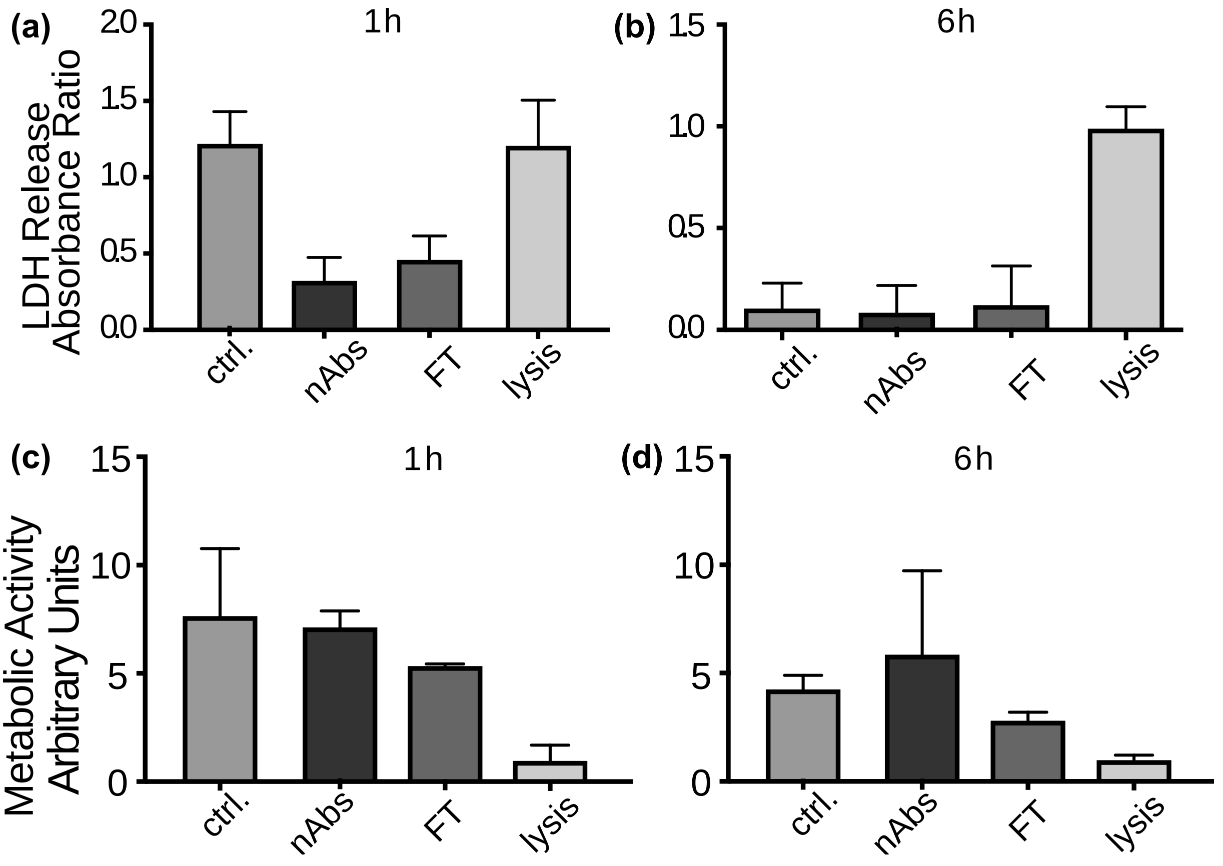

Supplementary Figure S2: nAbs-αSyn do not affect viability of untransfected HEK293T cells

Untransfected HEK293T cells were incubated with nAbs-αSyn, flow through (FT) or medium only (Ctrl.). LDH release (a, b) and metabolic activity (c, d) were measured after 1 h (a, c) and 6 h (b, d). Representative Graphs from three replicates, p values as calculated with one-way ANOVA.
